# Epidemiology of surgically treated posterior cruciate ligament injuries in Scandinavia

**DOI:** 10.1007/s00167-015-3786-2

**Published:** 2015-09-19

**Authors:** Christian Owesen, Stine Sandven-Thrane, Martin Lind, Magnus Forssblad, Lars-Petter Granan, Asbjørn Årøen

**Affiliations:** 1 Ortopedisk klinikk, Ahus, 1478 Nordbyhagen, Norway; 2 Nevrologisk avd, Ahus, 1478 Nordbyhagen, Norway; 3 Idrætssektoren, Ortopædkirurgisk Afd, Århus Sygehus, Tage Hansens Gade 2, 8000 Århus, Denmark; 4 Capio Artro Clinic, Stockholm Sports Trauma Research Center, Valhallavagen 91, 114 86 Stockholm, Sweden; 5 Senter for idrettsskadeforskning, Norges idrettshøgskole, Sognsveien 220, Postboks 4014 Ullevål Stadion, 0806 Oslo, Norway

**Keywords:** Knee, Posterior cruciate ligament, Knee ligament, Epidemiology, Knee registries

## Abstract

**Purpose:**

The main purpose of the study was to provide an overview of injury mechanisms, concomitant injuries, and other relevant epidemiological data for patients treated in Scandinavia with posterior cruciate ligament reconstruction (PCLR) following a posterior cruciate ligament (PCL) injury.

**Methods:**

A total number of 1287 patients who underwent PCLR from 2004 to 2013 in the Scandinavian counties were included from the national ligament registries. The variables such as age, sex, activity, and graft used for reconstruction were collected. Then, injuries were sorted based on concomitant injuries. Finally, data from the different registries were compared.

**Results:**

Average age of the treated patients was 32.7 years. Sex distribution ratio of male to female was 858:429 (66.7 %:33.3 %). Depending on definition, 26–37 % of the injuries treated were isolated PCL injuries. PCL injuries were most commonly encountered in sports with 35.4 % of the total number of PCL injuries in the study population. Soccer was the sport with the highest number of injuries (13.1 %). Cartilage lesions occurred in 26.1 % of PCL injuries and meniscal lesions in 21.0 %. Minimum one other additional ligament was injured in 62.2 %.

**Conclusion:**

Isolated PCL injuries are common, although the injury is most commonly associated with other ligament injuries. There is a high prevalence of cartilage injuries and meniscal lesions associated with PCL injuries. Sports are the leading cause of PCL injuries treated operatively. Epidemiological data are a necessary part of the basis for injury prevention in the future. The prevalence of concomitant injuries is also relevant and clinically important for the choice of surgical procedure and for the expected outcomes following surgery.

**Level of evidence:**

II.

## Introduction

The posterior cruciate ligament (PCL) is the stronger of the two cruciate ligaments in the knee and accounts for about 95 % of the total restrain to posterior translation of the tibia in regard to the femur [1]. In addition, the PCL has secondary stabilizing functions; it restraints rotation when the knee is flexed and remains in varus and valgus position when the knee is extended [2, 3].

The reported incidence of PCL injuries shows a great variation and is reported to be responsible for 1–44 % of all acute knee injuries [4]. This large variation might be due to some authors concentrating on trauma settings and others on the athletic population [5, 6]. There is also a variation in the report rate of isolated PCL injuries. Schulz et al. [6] reported that 47 % of the cases had isolated injuries and 53 % had concomitant injuries, according to the degree of posterior displacement (5–12 mm was classified as an isolated injury). Fanelli et al. [5, 7] on the other hand reported that the incidence of isolated injuries was 7.5 % and that 92.5 % was concomitant injuries (evaluated by arthroscopy). There is also some discrepancy when it comes to concurrent cartilage and meniscal lesions. Two previous studies describe observed cartilage lesions in about 30 % of the isolated PCL injuries [8, 9]. However, in a recent study, the reported incidence of cartilage injuries ICRS grade 3–4 was 9.9 % [10]. Geissler and Whipple [11] reported that out of 33 patients assumed to have an isolated PCL injury, 12 % also had cartilage defects and 27 % had meniscal tears.

The reported causes of PCL injuries are heterogeneous. Traditionally, the classic PCL injury is a result of a dashboard injury in traffic accidents, and traffic accidents have been considered a major cause of injuries to the PCL. Schulz found that 45 % of the PCL injuries were caused by motor vehicle accidents, and about 40 % were sports related. They also found that motorcycle accidents accounted for 28 % of the total PCL injuries and that soccer injuries accounted for 25 %. In soccer, the goalkeeper was most exposed to this type of injury [6]. Fanelli et al. [7] found that 56 % were trauma patients and 33 % were sports related. The most common pattern of injury is reported to be dashboard injuries and fall on the flexed knee with the foot plantar flexed [6].

It is clear that basic knowledge regarding aetiology of PCL injuries and their concomitant injuries is lacking. This fact makes it difficult to assess the representativeness of the different materials presented in the orthopaedic journals. The present study aims to present an unselected material of this knee ligament injury in order to cover this lack of knowledge in the literature. Since the Scandinavian cruciate ligament registries were established, there is only one published study focusing on the injured PCL [10]. Traditionally, PCL injuries have been treated nonoperatively, but this has over the years changed in favour of surgical reconstruction [12]. Since the Scandinavian registries include a high number of PCL reconstructions (PCLR), it is possible to make an analysis of injury mechanisms and concomitant injuries in those treated surgically.

## Materials and methods

The study design is a cross-sectional study on the activities leading to PCL injuries and concomitant injuries using data from the Scandinavian knee ligament registries. Patients were included from The Norwegian Knee Ligament Registry (NKLR), the Swedish Knee Ligament Registry (SKLR), and the Danish ACL Reconstruction Registry (DKRR). The NKLR was established in 2004 followed by the Swedish and Danish registries in 2005. The main objective of the NKLR was to prospectively register all surgical procedures on cruciate ligaments in Norway and to monitor the outcomes. Every hospital doing knee surgery in the Scandinavian countries reports knee ligament reconstructions to the respective registries. Both primary reconstructions and revision procedures are reported. The report rate to the Norwegian registry is approximately 86 % for anterior cruciate ligament (ACL) injuries with similar rates in Sweden and Denmark [13–15]. The registries contain no clinical information or grading of the PCL injuries. Information such as age, sex, activity leading to the injury, and any concomitant injury to the same knee is registered [16, 17]. A validated, self-reported knee outcome score form, The Knee Injury and Osteoarthritis Outcome Score (KOOS), is completed by the patients preoperatively and at follow-up on all patients at 1 or 2, 5, and 10 years post-operatively depending on country [16, 17]. In addition, both the Swedish registry and DKRR include EQ-5D, and DKRR also includes Tegner activity score. In Norway, informed consent is obtained from all patients for the preoperative KOOS, whereas this is not the case in Denmark and Sweden due to different legal requirements [18]. The surgeon completes a form post-operatively, with information regarding the findings and specifications of the performed procedure—including any concomitant injury to any other ligaments, menisci, joint cartilage, major nerve, and blood vessel injury. The cartilage injuries are graded according to the International Cartilage Repair Society (ICRS) grading scale 1–4 [19]. Any procedure to treat these injuries is also registered. The report rates to the respective registries have been fairly consistent in the registration period. When checked against each of the countries national patient registries, the report rates are about 90 %. The registries have been described in more detail in previous studies [15–17, 20].

**Fig. 1:**
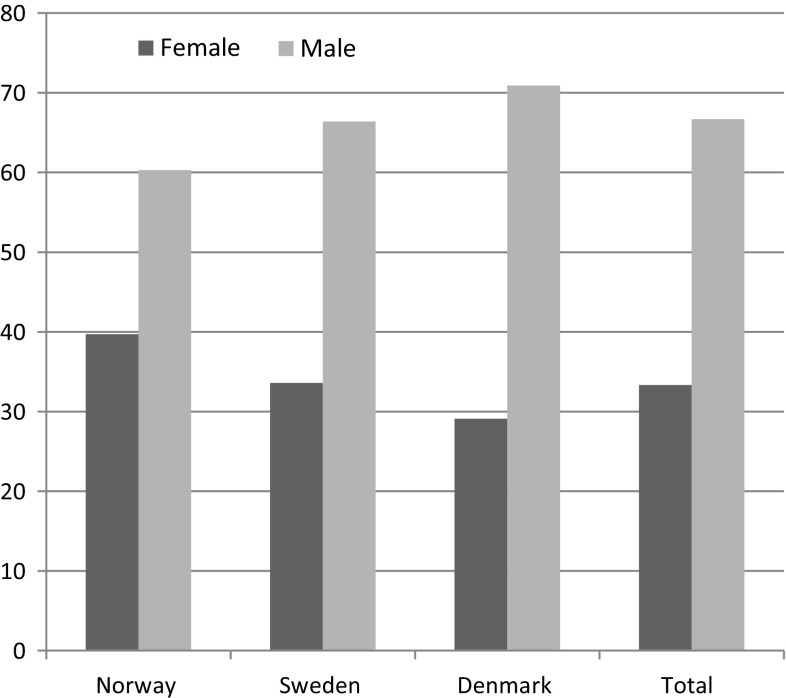
Sex distribution Scandinavian PCLR

For each of the registries, we calculated the patients mean age (Table 1), sex distribution (Fig. 1), and the number of the different grafts utilized and the total averages (Table 6). The patients were then sorted into groups (Table 5): isolated PCL injuries; PCL and other ligament injuries; PCL, other ligament injuries, and meniscal injuries; PCL, other ligament injuries, and cartilage injuries; PCL, other ligament injuries, cartilage injuries, and meniscal injuries; PCL and meniscal injuries; PCL and cartilage injuries; and PCL, meniscal injuries, and cartilage injuries. The injuries were sorted by the activities leading to the injuries (Table 3). Activities with quite high prevalence were kept separate, and activities with low prevalence (<1 %) were put together in joint categories. Corresponding data and variables for ACLR from the registries during the same period were used as a comparison to the PCLR data. Further, data regarding activity and concomitant injuries from the different registries were compared in order to look for differences and similarities between the three registries. The groups with the most obvious discrepancies were used to illustrate these differences.

**Table 1:** Age distribution PCLR

Sex	Norway avg.	Range	Sweden avg.	Range	Denmark avg.	Range	Total avg. age	Range
Female	32.6	14.2–67	30	12–62	32.7	15.6–59.9	31.9	12–67
Male	34.9	15–67	32	8–66	33.0	15.5–59.6	33.2	8–67
Total	34.0	14–67	31	8–66	32.9	15.5–59.9	32.7	8–67

### Ethics

Participation in the Norwegian and Swedish registries is voluntary for both surgeons and patients. Patients sign an informed consent, and in Norway, the NKLR is approved by the Norwegian Data Inspectorate. Similar rules and restrictions apply for the SKLR, although informed consent from the patients is not required. In Denmark, reporting to DKRR is mandatory for all clinics, and informed consent from the patients is not required. All data extracted from the registries are anonymized.

### Statistical analysis

The Statistical Analysis Product and Service Solutions (IBM Corp. Released 2011. IBM SPSS Statistics for Windows, version 20.0. Armonk, NY: IBM Corp) has been used to perform the descriptive analysis. The Chi-square test was used when comparing prevalence and the categorical data. Prevalence was calculated based on population size for the respective countries in the years 2004–2013, numbers provided by Wikipedia.

## Results

The total number of primary PCLR in the Scandinavian registries was 1287 in the years 2004–2013. The registries contain information on about 19,000 patients in Denmark, 17,000 patients in Norway, and 23,000 patients in Sweden during the same period. Among the PCLRs, there were two-thirds men and one-third women. The average age at the time of injury of the patients treated was 32.7 years (Table 1). The most frequent cause of PCL injury is sports with soccer as the largest contributor with. About one-fourth of the injuries was isolated PCL injuries (injury to no other structures injured registered), and in more than one-third of the reported cases, PCL was the only ligament injured (Table 5). The ligament most commonly injured together with the PCL was the ACL. A total of 270 patients had meniscal lesions and 337 had a cartilage injury ICRS grade 1–4. The most common graft used in reconstruction was hamstring autograft (Table 6).

**Fig. 2:**
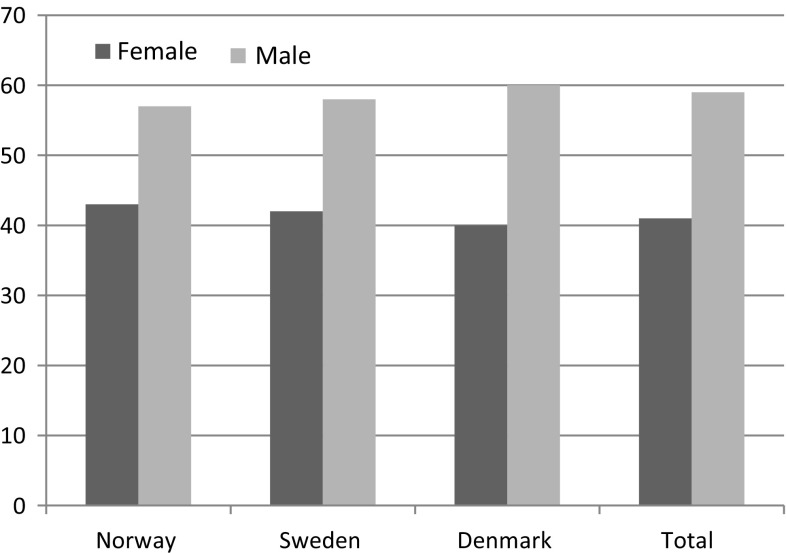
Sex distribution Scandinavian ACLR

For the ACLR patients, the average age was 28.5 years (Table 2). Male-to-female ratio was 60:40 (Fig. 2). The far most important activity causing the injuries was soccer. All sports in total account for about 80 % of the ACL injuries (Table 4). Compared to the ACLR group (Table 2), the PCLR patients are significantly older (*p* < 0.001). The male-to-female ratios are fairly similar with no significant differences. When it comes to the activity causing the injury, there are some differences. Football (soccer) is the single most common sports leading to both injuries (Tables 3, 4), but it accounts for a significantly higher number of the ACLRs compared to the PCLRs (*p* < 0.001). All sports in total account for a significantly higher percentage of the ACL injuries compared to the PCL injuries (*p* < 0.001). Traffic is a significantly more important cause of the PCL injuries (*p* < 0.001). There are also other categories with significant differences between the two types of injury, but the above mentioned are the most obvious.

**Table 2:** Age distribution ACLR

Sex	Norway avg.	Sweden avg.	Denmark avg.	Total avg. age
Female	25.8	27.0	29.6	27.8
Male	27.4	28.0	31.0	29.2
Total	26.7	27.6	30.5	28.7

**Table 3:** PCL injuries by activity

Activity	Norway	Sweden	Denmark	Total
Football (soccer)	38 (10.1 %)	51 (15.6 %)	79 (13.1 %)	168 (13.1 %)
Handball	30 (8.0 %)	14 (4.3 %)	36 (6.2 %)	80 (6.2 %)
Snowboard	6 (1.6 %)	2 (0.6 %)	1 (0.2 %)	9 (0.7 %)
Alpine skiing (incl. twin tip)	33 (8.8 %)	33 (10.1 %)	35 (6.2 %)	101 (7.8 %)
Other ski activity	58 (15.5 %)	2 (0.6 %)	1 (0.2 %)	61 (4.7 %)
Martial arts	4 (1.1 %)	7 (2.1 %)	2 (0.3 %)	13 (1.0 %)
Team sports (ice hockey, bandy, etc.) inline skating volleyball, basket,	6 (1.6 %)	15 (4.3 %)	4 (0.7 %)	25 (1.9 %)
Motorsport and car sport including traffic	81 (21.6)	102 (31.2 %)	199 (34.0 %)	382 (29.7 %)
Other physical activity (other sports, dancing, etc.)	53 (14.1 %)	41 (12.5 %)	74 (12.6 %)	168 (13.1 %)
Work related	22 (5.9 %)	19 (5.8 %)	40 (6.8 %)	81 (6.3 %)
Fall, jumping, play including trampoline and skateboard	21 (5.6 %)	3 (0.9 %)	0 (0 %)	24 (1.9 %)
Outdoor recreation	7 (1.9 %)	10 (3.1 %)	0 (0 %)	17 (1.3 %)
Other	6 (1.6 %)	27 (8.3 %)	75 (12.8 %)	108 (8.4 %)
Missing/unknown	10 (2.7 %)	1 (0.3 %)	39 (6.7 %)	50 (3.9 %)
Total	375 (100 %)	327 (100 %)	585 (100 %)	1287 (100 %)

Numbers and percentages for each country and total

**Table 4:** ACL injuries by activity

Activity	Norway	Sweden^a^	Denmark	Total
Football (soccer)	7043 (40.1 %)	6470 (42.0 %)	7928 (40.4 %)	21,441 (41.1 %)
Handball	2504 (14.3 %)	760 (4.7 %)	3186 (16.3 %)	6450 (12.4 %)
Snowboard	395 (2.3 %)	156 (0.6 %)	68 (0.4 %)	619 (1.2 %)
Alpine skiing (incl. twin tip)	2194 (12.5 %)	1850 (14.4 %)	2406 (12.3 %)	6450 (12.4 %)
Other ski activity	443 (2.5 %)	13 (0.1 %)	28 (0.1 %)	484 (0.9 %)
Martial arts	330 (1.9 %)	356 (2.7 %)	173 (0.9 %)	859 (1.6 %)
Team sports (ice hockey, bandy, etc.) inline skating volleyball, basket,	494 (2.8 %)	2126 (13.4 %)	256 (1.3 %)	2876 (5.5 %)
Motorsport and car sport including traffic	405 (2.3)	574 (3.6 %)	615 (3.1 %)	1594 (3.1 %)
Other physical activity (other sports, dancing, etc.)	993 (5.7 %)	993 (7.8 %)	1973 (10.1 %)	3959 (7.6 %)
Work related	436 (2.5 %)	267 (1.8 %)	550 (2.8 %)	1253 (2.4 %)
Fall, jumping, play including trampoline and skateboard	753 (4.3 %)	115 (1.2 %)	1619 (8.3 %)	2487 (4.8 %)
Outdoor recreation	0 (0.0 %)	185 (1.4 %)	0 (0.0 %)	185 (0.4 %)
Other	1150 (6.6 %)	1106 (6.3 %)	0 (0.0 %)	2256 (4.3 %)
Missing/unknown	409 (2.3 %)	0 (0.0 %)	804 (4.1 %)	1213 (2.3 %)
Total	17,549 (100 %)	14,971 (100 %)	19,606 (100 %)	52,126 (100 %)

Numbers and percentages for each country and total

^a^2005, 2008, 2009, 2012, and 2013. Data from 2006, 2007, 2010, and 2011 are not available

There was a higher prevalence of PCLRs performed among the total national population from 2004 to 2013 in Denmark 10.6/100.000, 95 % confidence interval CI (8.0, 13.2) and Norway 7.4/100.000, CI (4.6, 10.2) compared to Sweden 3.6/100.000, CI (1.5, 5.7). The differences between the countries are statistically significant (*p* < 0.001). There was also a statistically significant higher prevalence of cartilage lesions in Norway 37.3 %, 95 % CI (32.4, 42.2) and Sweden 37.8 %, CI (32.5, 43.1) compared with Denmark 12.5 %, CI (9.8, 15.2), (*p* < 0.001). Among the PCLRs, there was also a statistically significant higher prevalence of meniscal lesions in Norway 24.2 %, CI (19.9, 28.5) and Sweden 23.5 %, CI (18.9, 28.1) compared to Denmark 17.4 %, CI (14.3, 20.5), (*p* < 0.001).

## Discussion

The main findings of this study were that the number of isolated PCL injuries account for about one-third of the total number of PCL injuries (Table 5). This is new information regarding knee ligament injuries. Isolated PCL injuries are therefore clinically important. Despite this, injuries to the PCL most often appear together with other ligament injuries, where a combination with ACL is the most common. PCL injuries together with meniscal or cartilage lesions, but no other ligament injury, are quite rare, each accounting for 3.0 and 6.5 %, respectively, and 1.9 % with combination of both meniscal and cartilage lesions. Meniscal and cartilage injuries are usually seen when there are other ligament injuries accompanying the PCL injury. They both appear in similar frequencies (Table 5). This can be explained by the injury mechanism involving forces with a higher amount of energy causing the injury. An isolated PCL injury often occurs as a result of a dashboard injury, fall on flexed knee, or hyperextension of the knee as is shown by anatomical and biomechanical studies focusing on the stabilizing function of the PCL [12, 21–23]. PCL injuries in combination with another ligament injury are more likely when the mechanism of injury contains a rotational component and/or valgus/varus stress. Meniscal and cartilage lesions are also more likely to occur when there are rotational forces and/or varus and valgus stress involved [24–26]. One could speculate that there is some degree of relation between the injury mechanism and the concomitant injuries.

**Table 5:** Combinations of injuries

Injured structures	Norway	Sweden	Denmark	Total
PCL	69 (18.4 %)	82 (25.1 %)	189 (32.3 %)	340 (26.4 %)
PCL + other ligament	121 (32.3 %)	86 (26.3 %)	246 (42.1 %)	453 (35.2)
PCL + other ligament + cartilage + meniscus	39 (10.4 %)	31 (9.5 %)	18 (3.1 %)	88 (6.8 %)
PCL + meniscus	7 (1.9 %)	9 (2.6 %)	22 (3.8 %)	38 (3.0 %)
PCL + cartilage	26 (6.9 %)	42 (12.8 %)	16 (2.7 %)	84 (6.5 %)
PCL + meniscus +cartilage	7 (1.9 %)	11 (3.4 %)	7 (1.2 %)	25 (1.9 %)
PCL +other ligament + cartilage	68 (18.1 %)	40 (12.2 %)	32 (5.5 %)	140 (10.9 %)
PCL + other ligament + meniscus	38 (10.1 %)	26 (8.0 %)	55 (9.4 %)	119 (9.2 %)
Total	375 (100 %)	327 (100 %)	585 (100 %)	1287 (100 %)
Total PCL + min 1 other ligament	266 (70.1 %)	183 (60.0 %)	351 (60.0 %)	800 (62.2 %)
Tot. PCL + meniscus	91 (24.2 %)	77 (23.5 %)	102 (17.4 %)	270 (21.0 %)
Tot. PCL + cartilage	140 (37.3 %)	124 (37.9 %)	73 (12.5 %)	337 (26.1 %)
Tot. PCL without other ligament	108 (28.8 %)	144 (44.0 %)	234 (40.0 %)	486 (37.8 %)

Numbers and percentages for each county and total

The distribution of activity shows that almost one-third of the PCL injuries was related to vehicle accidents or motorsports accidents. Football (soccer) and skiing activities were the most important sports activities leading to a PCL injury. Other physical activity (like dancing and some team activities) was also an important category (Table 3). This is in some contrast to classical teaching that clearly states that PCL injuries are almost exclusively the result of traffic accidents. Importantly, the numbers are even more in favour of sports if motorsports are taken away from the traffic category. One can argue that motorsports is not traffic since normal traffic rules do not apply, and it is performed under different circumstances than usual traffic. This finding is in some contrast to the assumption that PCL injuries result from traffic accidents [7], but corresponds to findings in other studies [4, 6].

There are some differences in the activities leading to the injuries between the respective countries. When it comes to injuries in motorsports and traffic, this is more commonly seen in Sweden and Denmark than in Norway. The difference between Norway and Sweden could theoretically be explained by the difference in licenced competitors of the sports with close to 23.000 members in Norway (Norsk bilsportsforbund) and about 120.000 in Sweden (Svensk bilsport), but in Denmark there are only about 8.000 licenced competitors (Danks bilsport). However, there is another possible explanation. There was a higher average number per year of seriously injured people in traffic accidents registered in Sweden and Denmark compared to Norway in the years 2004–2012. The numbers for injuries classified as serious were 2689 in Denmark, 1122 in Sweden (numbers available only 2007–2012 for Sweden), and 825 in Norway. These numbers include all injuries classified as serious and not only knee injuries. However, the numbers provide information on how many people are injured in traffic and might say something about the probability of a traffic-related PCL injury. As one might expect, skiing activities (including snowboard) are more common in Sweden and Norway compared to Denmark, as there is only one small ski centre in the whole of Denmark where there are several in both Sweden and Norway.

The graft choices in the registries reflect some difference in practice between the Scandinavian countries (Table 6) and can perhaps be explained by the accessibility of allografts and traditions for using different types of grafts. Denmark is geographically a much smaller country than Sweden and Norway. A higher number of PCL reconstructions are performed at a few referral hospitals, whereas in Norway and Sweden some hospitals perform as few as one or two PCLRs per year. With a higher number of reconstructions, it is easier to obtain allografts and have good procedures performing reconstructions with these grafts. There is a lower prevalence of PCLRs in Sweden compared to the neighbouring countries. One could speculate that this is due to a lower report rate, but this is supposedly not the case as the report rate has been confirmed to be about 90 % for ACLR [13]. As the SKLR was mainly planned as an ACL registry, it could be that there is a lower report rate for PCLR, although this is not known and needs to be further investigated. This leaves two possibilities: that there in fact are fewer occurring PCL injuries in Sweden, or that a lower number of these are treated operatively. Why this remains unclear. There are a lower number of meniscal and cartilage injuries among the Danish PCLR patients. This might be partly due to a higher prevalence of PCL injuries without any other ligament injury in their population, but exactly why this still remains unclear.

**Table 6:** Graft choices

Graft	Norway	Sweden	Denmark	Total
Hamstring	257 (68.5 %)	157 (48.0 %)	237 (40.5 %)	651 (50.6 %)
Allograft	42 (11.2 %)	49 (15.0 %)	197 (33.7 %)	288 (22.4 %)
Patellar tendon	25 (6.7 %)	5 (1.5 %)	9 (1.5 %)	39 (3.0 %)
Direct suture	7 (1.9 %)	22 (6.7 %)	1 (0.2 %)	30 (2.3 %)
Quadriceps	30 (8.0 %)	75 (27.8 %)	141 (24.1 %)	246 (19.1 %)
Unknown	14 (3.7 %)	19 (5.8 %)	0 (0 %)	33 (2.6 %)
Total	375 (100 %)	327 (100 %)	585 (100 %)	1287 (100 %)

Numbers and percentages for each country and total

The difference in age for the ACL and PCL patients is similar to what has been found in a previous study [10]. The reason for this difference remains unknown but can possibly partly be explained by a higher number of sports injuries in the ACL group and a higher number of traffic injuries responsible for the PCL injuries. The reason for traffic causing relatively more PCL injuries than ACL injuries is probably related to the injury mechanism with a direct blow against the tibia. The energy involved in traffic accidents is also often higher than in sports injuries. This is relevant information when we know that more energy is needed to tear the PCL than the ACL.

Strengths of this study are that the registries contain information on activity and concomitant injuries. There are a limited number of studies on injury mechanisms and concomitant injuries. Most of the studies in the literature either have small numbers of patients or have focused on trauma patients. Therefore, it is likely that neither of the published studies reflects the true PCL injured population. In the Scandinavian registries, all types of injuries are included from a large geographical area. This provides a more representative estimate than those previously published when it comes to surgically treated PCL injuries. Simultaneously, there are known limitations when using registry data. Nonoperative treatment is an alternative for both ACL and PCL injuries [4, 27]. Information on patients treated nonoperatively is not included in the registries. Objective clinical information is sparse. The registries are not complete, and we do not know for sure how the missing data could affect the results of this study. There could also be underreporting of concomitant injuries by the surgeons as some injuries are easily missed on MRI or by the individual surgeon. This specifically applies to injuries to the posterolateral corner. Only a minority of the total number of patients have undergone stress radiographs, as this is so far only recorded in the DKRR. Another limitation is that this study reflects the Scandinavian population. It is not clear whether findings in other countries will be comparative as there are differences even between the Scandinavian countries.

Sports are the leading cause of PCL injuries treated operatively in the study population. Epidemiological data are a necessary part of the basis for injury prevention in the future. Increased focus on PCL injuries in sports may lead to interventions aiming to reduce the frequencies of the injuries. The prevalence of concomitant injuries is also relevant and clinically important for the expected outcomes following surgery. It is also important when considering where to treat these patients, as some of the concomitant injuries often require what is usually considered technically demanding surgery. PCL reconstruction should probably be performed in regional hospitals with experienced surgeons used to this type of injuries.

## Conclusion

Patients undergoing PCLR in the Scandinavian countries often have other related injuries to the same knee, although isolated PCL injuries are common. The PCL is most commonly injured in sports. The registries in the different countries show some differences in the prevalence of PCLRs and related injuries. The activity leading to the injuries is fairly similar in the different countries with some expected differences, skiing activities are more common causes in Norway and Sweden than Denmark, and traffic including motorsports is more common in Sweden and Denmark compared to Norway. Sports is a more frequent cause of PCL injuries than frequently presented in the literature, and this clinically important information has to be taken into account when assessing the representativeness of research on PCL injuries or other knee injuries involving a PCL injury.
